# Correlation analysis of hysterectomy and ovarian preservation with depression

**DOI:** 10.1038/s41598-023-36838-2

**Published:** 2023-06-16

**Authors:** Yunhong Yang, Xiangqi Zhang, Yinuo Fan, Jiahao Zhang, Bingchun Chen, Xiaofeng Sun, Xiaofeng Zhao

**Affiliations:** 1 Guangzhou University of Chinese Medicine, Guangzhou, China; 2 Taihe Town Health Center, Baiyun District, Guangzhou, China; 3 The First Affiliated Hospital, Guangzhou University of Chinese Medicine, Guangzhou, China

**Keywords:** Psychology, Medical research, Clinical trial design

## Abstract

The relationship between hysterectomy and ovarian preservation and depression is controversial. This study aimed to determine the association of hysterectomy and ovarian preservation with depression using National Health and Nutrition Examination Survey. To assess the association between hysterectomy with or without ovariectomy and depression, we used 3 methods. Method 1: propensity score model (PSM) was established. Method 2 was logistics regression analysis of hysterectomy and depression before and after PSM. Method 3 was a logistics regression analysis of the relationship between hysterectomy and different depressive symptoms. At the same time, in order to evaluate the association between hysterectomy with or without oophorectomy and depression, we explored the effect of four different surgical procedures on depression using logistic regression equations. We enrolled 12,097 women, of whom 2763 underwent hysterectomy, 34.455% were positive for depression. After weighting, 33.825% of the total sample had a PHQ ≥ 5. Finally, a total of 2778 women were successfully matched by propensity score, and 35.537% of them were positive for depression. The OR for PHQ ≥ 5 was 1.236 after crude adjustment of covariates and 1.234 after exact adjustment. This suggests that Hysterectomy is strongly associated with positive depression. Positive depression (PHQ ≥ 5) was associated with little interest, feeling down and trouble concentrating. It was not associated with trouble sleeping, feeling tired, poor appetite, feeling bad, slow moving or speaking, and suicidal thoughts. Oophorectomy-alone is not associated with depression. Hysterectomy-alone is a risk factor for depression, but Hysterectomy combined with Oophorectomy has a stronger correlation with depression than Hysterectomy-alone. Women who have had a Hysterectomy are at higher risk of depression than women who have not had a Hysterectomy, and this risk may be exacerbated if the uterus and ovaries are removed. When clinically appropriate, surgeons should try to preserve the patient's ovaries.

## Introduction

Hysterectomy is a frequently performed gynecological procedure^1^, primarily indicated for perimenopausal uterine fibroids, adenomyosis, and other conditions with high recurrence rates. Uterine fibroids and adenomyosis are most prevalent in women aged 45–49 years^2^. In view of uterine aging and patients' fear of malignant transformation of leiomyoma, some scholars suggest that patients with uterine fibroids over 40 years old directly undergo hysterectomy^3^. A study involving 227,489 patients with uterine fibroids revealed a 4.1% likelihood of receiving myomectomy followed by hysterectomy^4^.Studies have shown that the incidence of hysterectomy is 11%^5^. The commonly used surgical methods include Hysterectomy and salpingectomy, or Hysterectomy and monoliteral or bilateral adnexectomy. Currently, total hysterectomy with or without oophorectomy is a common clinical practice for genital lesions such as uterine fibroids, adenomyoma, functional bleeding, and benign ovarian cysts. Studies in developed countries have shown that 20–40% of women undergo hysterectomy by the age of 60 years^6,7^, with bilateral ovaries removed at the same time in 10–55% of cases^8^. However, while hysterectomy effectively treats gynecological physiological diseases, it also gives rise to psychological issues that trouble patients.

Since the proposal of “post-hysterectomy syndrome” in the 1970s, an increasing number of clinical studies have demonstrated^9–11^ that women who have undergone hysterectomy are more susceptible to psychological comorbidities such as insomnia, anxiety, and depression than those who have not. Depression is a prevalent psychological disease characterized by persistent and significant low mood, causing immense physical and mental distress to patients. It has emerged as the third leading cause of global disease burden^12^. According to a survey conducted by the World Health Organization in 2015, approximately 322 million individuals worldwide were afflicted with depression^13^. Between 2013 and 2016, an estimated 8.1% of adults experienced symptoms of depression within a 2-week period^14^. In recent years, epidemiological research has revealed significant gender disparities in the prevalence, incidence, course, symptoms and risk factors of depression^15,16^. Current research indicates that the prevalence of depression is twice as high in women compared to men^17,18^, However, some studies have suggested that hysterectomy alone does not increase the risk of depression^19^, and a positive correlation may be observed when combined with ovariectomy^20,21^. Conversely, other studies suggest that ovarian preservation may actually elevate the likelihood of developing depression^22^.

The relationship between hysterectomy and ovarian preservation and depression remains a topic of debate. Therefore, the purpose of this study was to investigate the potential association between these procedures and depression using data from the National Health and Nutrition Examination Survey.

## Data and methods

### Study population

**Figure 1:**
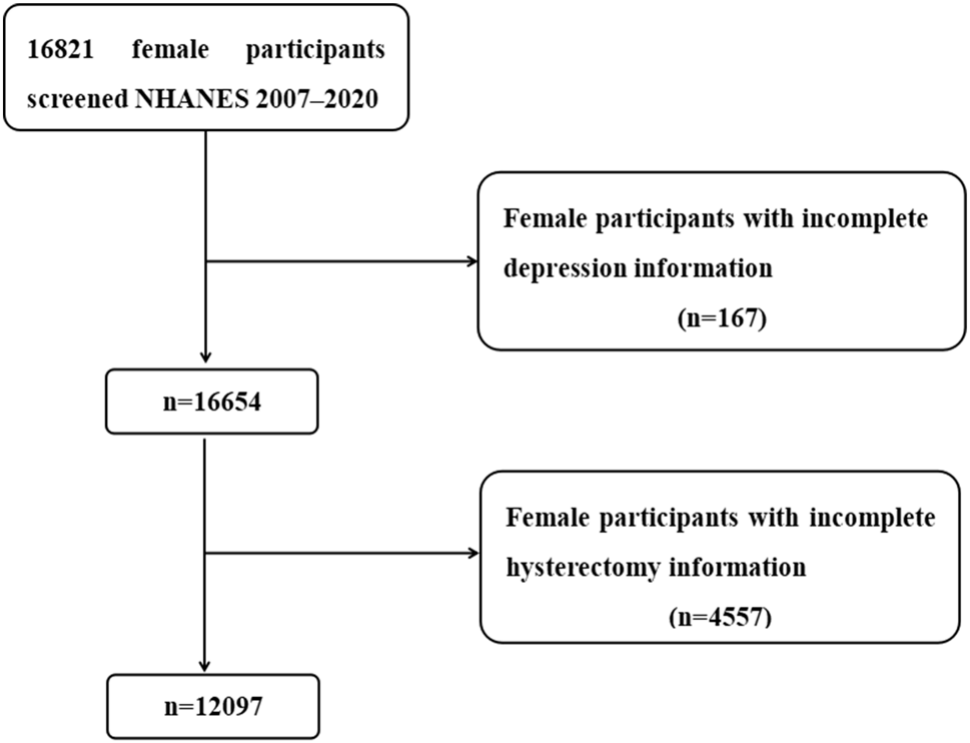
Flowchart of screening samples from NHANES.

The National Health and Nutrition Examination Survey (NHANES) is a sophisticated multi-stage sampling design that selects samples to assess health and dietary status of civilian and non-institutional populations in the United States every 2 years, with resulting data publication^23,24^. In this study, participants were drawn from continuous, cross-sectional NHANES data from 2007 to 2020 in the United States. We included a total of 16,821 women ages 18 and older who responded to reproductive health questions about hysterectomy and mental health questions pertaining to depression using Mobile Screening Center (MEC). Excluding women with incomplete information on depression or incomplete information on hysterectomy. In the end, a total of 12,097 women participated in our study (Fig. 1). The study was approved by the NCHS Research Ethics Review Committee (https://www.cdc.gov/nchs/nhanes/irba98.htm), regulations and the written informed consents were obtained from all participants and all experiments were performed in accordance with relevant guidelines.

### Hysterectomy and oophorectomy

Hysterectomy was identified by RD280 (Had a hysterectomy?) in the self-report question and oophorectomy by RHQ305 (Had both ovaries removed?), both of which were part of the 2007–2020 National Reproductive Health Questionnaire.

### Outcome indicator

Participants were assessed for depressive symptoms using the health Questionnaire (PHQ-9), which has good reliability and can effectively screen for depression or depressive symptoms in the past 2 weeks. The questionnaire consisted of nine questions, each of which was rated on a four-point scale from 0 to 3, with a score indicating the frequency of symptoms. The score ranges from 0 to 27. The nine diagnostic items included little interest, feeling down, trouble sleeping, poor appetite, feeling tired, feeling bad, trouble concentrating, moving or speaking slowly, suicidality and behavior. Participants with an overall score of 5 and above are considered to be positive for depression, with 5 being the PHQ threshold^25^.

### Covariate

Based on previous literature and the availability of NHANES data, a number of potential confounding factors were included in this analysis. (a) demographic and socioeconomic status, including age, race/ethnicity, education level, poverty income ratio, body mass index(BMI), marital status, smoking history, alcohol consumption. (b) Past history including surgical history including ovariectomy, chronic diseases about hypercholesterolemia, diabetes, hypertension, weak or failing Kidneys and trouble sleeping, drug use includes the use of female hormones (Table 1).

**Table 1:** Characteristics of participants including general characteristics and past medical histories (n%).

Characteristics	Propensity overlap weighting			Propensity 1:1 matching			Propensity overlap weighting after psm		
	Q1	Q2	*P* value	Q1	Q2	*P* value	Q1	Q2	*P* value
Age (years)%			< 0.00001			< 0.001			< 0.00001
< 30	4.457	0.103		5.832	0.216		6.87	0.213	
30–44	6.399	1.878		15.695	1.944		19.045	2.209	
45–54	4.085	4.506		11.663	4.968		15.371	6.121	
55–64	2.45	4.685		14.039	5.112		13.822	4.179	
≥ 65	2.154	7.897		19.366	9.503		13.293	6.803	
Missing	80.455	80.932		33.405	78.258		31.6	80.475	
BMI	28.007 ± 7.077	29.271 ± 6.676	< 0.00001	29.558 ± 7.284	29.831 ± 6.843	0.31	29.065 ± 7.158	29.291 ± 6.638	0.38887
Race (%)			< 0.00001			0.439			0.93634
Hispanic and others	21.464	12.292		28.15	28.078		14.632	14.854	
Non-hispanic White	66.924	75.622		46.436	44.492		70.935	70.325	
Non-hispanic Black	11.611	12.087		25.414	27.430		14.433	14.821	
Education level (%)			< 0.00001			0.002			0.00102
< High school	14.21	17.838		34.845	28.654		23.273	20.514	
High school	19.53	26.169		23.326	24.406		24.979	25.975	
Some college	33.741	35.833		25.990	31.461		29.004	35.285	
> College graduate	32.488	20.102		15.839	15.407		22.744	18.208	
Missing	0.03	0.057		0.000	0.072		0.00	0.018	
PIR (%)			< 0.00001			0.207			0.00337
< 1.0	15.891	11.916		22.606	20.662		13.745	13.691	
1.0–2.0	20.031	23.704		26.782	29.518		18.996	24.16	
≥ 2.0	60.733	60.479		50.612	49.820		67.258	62.15	
Missing	3.344	3.902					0.00	0.00	
Marital status (%)			0.73867			< 0.001			< 0.00001
Married or living with partner	39.691	40.052		40.461	50.468		30.511	40.692	
Widowed or divorced or separated	60.309	59.948		59.539	49.532		69.489	59.308	
Smoked at least 100 cigarettes in a lifetime	37.375	45.518	< 0.00001	39.021	43.053	0.031	41.254	45.239	0.03399
Alcohol consumption (%)	70.823	62.816	< 0.00001	55.22	56.587	0.468	61.969	63.775	0.3247
Ovariectomy (%)	0.339	53.4	< 0.00001	2.448	7.127	< 0.001	2.466	7.207	< 0.00001
Hormone use (%)	7.121	5.695	< 0.00001	4.68	3.528	< 0.001	6.863	4.293	< 0.00001
Hypertension (%)	26.499	56.308	< 0.00001	59.467	58.459	0.503	53.664	53.173	0.9665
Hypercholesterolemia (%)	26.974	51.81	< 0.00001	39.813	49.028	< 0.001	39.354	46.668	< 0.00001
Diabetes (%)	7.339	16.278	< 0.00001	18.143	21.598	0.029	12.748	15.331	0.14522
Weak or failing Kidneys (%)	2.15	4.86	< 0.00001	2.880	4.752	0.01	2.452	3.361	0.15389
Trouble sleeping (%)	28.283	43.959	< 0.00001	31.030	39.597	< 0.001	31.504	41.775	< 0.00001
Depression PHQ ≥ 5	25.934	33.825	< 0.00001	31.102	36.645	0.002	27.861	35.537	0.00001

Significance at *P* < 0.05.

Q1: Non-hysterectomy, Q2: Hysterectomy.

### Statistical method

#### Descriptive statistics

The data are reported as mean ± SD and Min–Max for continuous variables and percentages for categorical variables. The normality of continuous variables was assessed using the Shapiro–Wilk test. If the data followed a normal distribution, statistical significance was determined by means of Student's t-test. In cases where non-normal distribution was observed, Kruskal–Wallis test was employed to determine statistical significance. Due to the intricate design of NHANES, it is imperative to utilize appropriate weights while estimating data that represents the deinstitutionalized civilian population of the United States. For this particular study, subsample B's weight has been employed.

#### Model

To assess whether there is an association between hysterectomy and depression, we used the following methods.

Method 1: Establishment of a propensity score model (PSM).

The propensity score model (PSM) is a non-parsimonious multivariate logistic regression model^26^, and propensity score covariates can be found in Table 1. The data of the two groups were matched with propensity score, nearest neighbor matching method was adopted, caliper value was set as 0.2, and the two groups were matched according to 1:1^27^. After PSM, the distribution of covariates reached equilibrium among groups (*P* > 0.05). The PSM model was built using the statistical software IBM SPSS Statistics 25.0.

Method 2 was logistic regression analysis of hysterectomy and depression.

After obtaining PSM data, we used logistic regression to analyze the relationship between hysterectomy and the dichotomous depression measure^25^ and each depressive symptom^28^, respectively, as well as to analyze the effect on depression with or without ovariectomy. First, regression analysis was performed for depression positive or negative (Table 2). Second, to explore the relationship between independent variables and depressive symptoms, three models were designed (Table 3). Model 1 represents unadjusted outcomes. Model 2 is a coarsely adjusted logistic regression after propensity score matching, adjusting for age, marital status, poverty-income ratio, education level, smoking history, ovariectomy status, female hormone use, hypercholesterolemia and sleep disturbance. Model 3 is an adjustment for all covariables.

**Table 2:** Unadjusted, crude and adjusted odds ratios (95% confidence intervals) for positivity of depression after hysterectomy.

	N	Odds ratios (95% CI)					
		Unadjusted	*P* value	Crude	*P* value	Adjusted	*P* value
Propensity overlap weighting before psm	12,097	1.307 (1.194, 1.431)	< 0.001	1.145 (1.004, 1.306)	0.04	1.128 (0.987,1.289)	0.08
Propensity overlap weighting after psm	2778	1.281 (1.095, 1.500)	0.002	1.236 (1.016, 1.505)	0.03	1.234 (1.007, 1.512)	0.04

a. Crude: Adjusted for marital status, PIR, education level, smoking history, oophorectomy, female hormone use, hypercholesterolemia, and sleep disorders. b. Adjusted: Adjusted for all the covariates.

**Table 3:** β (95% CIs) of hysterectomy associated with depressive symptoms.

	Model 1 β (95% CI)	Model 2 β (95% CI)	Model 3 β (95% CI)
Little interest	0.128 (0.071, 0.185)	0.076 (0.010, 0.142)	0.084 (0.018, 0.150)
Feeling down	0.074 (0.016, 0.133)	0.062 (− 0.005, 0.129)	0.069 (0.002, 0.136)
Trouble sleeping	0.028 (− 0.048, 0.103)	0.031 (− 0.053, 0.116)	0.034 (− 0.050, 0.119)
Feeling tired	0.082 (0.009, 0.155)	0.018 (− 0.065, 0.100)	0.038 (− 0.044, 0.120)
Poor appetite	0.082 (0.018, 0.146)	0.036 (− 0.039, 0.111)	0.048 (− 0.026, 0.123)
Feeling bad	− 0.005 (− 0.056, 0.047)	0.016 (− 0.044, 0.076)	0.018 (− 0.042, 0.079)
Trouble concentrating	0.093 (0.040, 0.145)	0.080 (0.020, 0.140)	0.087 (0.026, 0.147)
Moving/speaking slowly	0.044 (0.003, 0.086)	0.039 (− 0.009, 0.087)	0.040 (− 0.008, 0.088)
Better off dead	0.019 (− 0.003, 0.041)	0.009 (− 0.017, 0.035)	0.009 (− 0.018, 0.035)

Model 1 represents the unadjusted outcome. Model 2 was adjusted for marital status, PIR, education level, smoking history, oophorectomy, female hormone use, hypercholesterolemia, and sleep disorders. Model 3 refers to the adjustment for all covariates. A: Hysterectomy without Oophorectomy. B: Oophorectomy without Hysterectomy. C: Non-hysterectomy without Oophorectomy. D: Hysterectomy with Oophorectomy.

**Figure 2:**
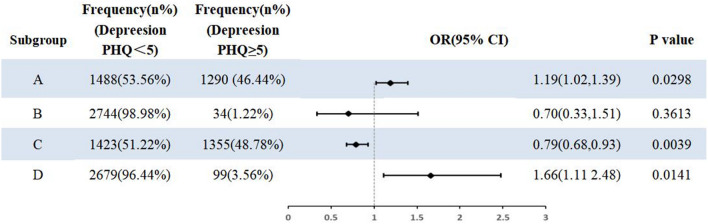
Logistics regression analysis of different surgical methods on positive depression.

Finally, in order to evaluate the correlation between the preservation of the fallopian tube ovary and depression, logistic regression was used to analyze the relationship between different surgical methods and depression (Table 4). Odds ratios were obtained by adjusting for covariates. Forest plots were drawn to visualize the data (Fig. 2).

**Table 4:** The distribution of patients with depression by four different surgical procedures.

	Subgroup A		Subgroup B		Subgroup C		Subgroup D	
	Yes (N = 1290)	No (N = 1488)	Yes (N = 34)	No (N = 2744)	Yes (N = 1355)	No (N = 1423)	Yes (N = 99)	No (N = 2679)
Depression PHQ ≥ 5 (N%)	464 (35.97%)	477 (32.06%)	9 (26.47%)	932 (33.97%)	423 (31.22%)	518 (36.40%)	54 (54.55%)	896 (33.45%)
Depression PHQ < 5 (N%)	826 (64.03)	1011 (67.94%)	25 (73.53%)	1812 (66.03%)	932 (68.78%)	905 (63.60%)	45 45.45%)	1783 (66.55%)
*P* value	0.030		0.359		0.004		0.013	

Subgroup A: Hysterectomy without Oophorectomy. Subgroup B: Oophorectomy without Hysterectomy. Subgroup C: Non-hysterectomy without Oophorectomy. Subgroup D: Hysterectomy with Oophorectomy.

All logistic analyses were performed with R software, V 0.4.0.3 [R: a language and statistical computing environment (program). Vienna, Austria: R Foundation for Statistical Computing, 2016], and EmpowerStats (http://www.empowerstats.com). The figures were generated using Adobe Photoshop (https://www.adobe.com/products/photoshop.html) or Origin 2021 (https://www.originlab.com/). Finally, we confirm that all methods were carried out in accordance with relevant guidelines and regulations.

## Result

### Descriptive statistical analysis

Prior to propensity score matching, 2763 of 12,097 women underwent hysterectomy, of whom 952 were positive for depression, accounting for 34.455% of the sample (*P* < 0.001). The sample was weighted so that patients with PHQ ≥ 5 points accounted for 33.825% of the total sample (*P* < 0.001). By adjusting for covariates for propensity matching, a total of 2778 women in the database were successfully matched to the hysterectomized and non-hysterectomized population of 1389 each, with 35.537% of the sample in the hysterectomized group experiencing depression (*P* < 0.001). And 35.537% were positive for depression in the hysterectomy group.

### Logistic regression

A PHQ score of 5 was used as the cut-off point for the presence or absence of depression. Before propensity score matching, the OR for PHQ score of 5 or more was 1.145 (95%CI 1.004, 1.306) after crude adjustment of covariates, and 1.128 (95%CI 0.987, 1.289) after exact adjustment. In 2778 subjects after propensity 1:1 matching, the OR for PHQ ≥ 5 was 1.236 (95%CI 1.016, 1.505) after crude adjustment of covariates and 1.234 (95%CI 1.007, 1.512) after exact adjustment. This suggests that hysterectomy is associated with positivity for depression.

To further explore the key to hysterectomy and depression, we performed a regression analysis for each depressive symptom. The results showed that positive depression was related to little interest and trouble concentrating. It was not associated with trouble sleeping, feeling tired, poor appetite, feeling bad, slow moving or speaking, and suicidal thoughts. Oophorectomy alone is not associated with depression.

In addition, we distinguish in detail between four different types of surgery, including Hysterectomy without Oophorectomy, Oophorectomy without Hysterectomy, Non-hysterectomy without Oophorectomy, and Hysterectomy with Oophorectomy. The number of depressed patients in each group was 464,9,423, and 54, respectively. Visualizations were plotted with adjusted covariates. Hysterectomy alone is a risk factor for depression, but hysterectomy combined with oophorectomy has a stronger correlation with depression than hysterectomy alone.

## Discussion

At present, gynecological malignancies^29^, endometrial hyperplasia with dysplasia^30,31^, intractable postpartum hemorrhage^32,33^, or prophylactic resection with a family history of tumors are suitable diseases for hysterectomy. Due to the differences in individualization between patients, surgeons need to perform hysterectomy according to professional knowledge, indications for surgery, nature of the disease^34^, patient characteristics and patient willingness. However, psychosocial problems after hysterectomy should not be ignored. Post-hysterectomy syndrome makes researchers raise the concern about postoperative complications. Gupte and Nagabhirava found that 9% of women had post-operative depression^35^, of which 2% were post-operative new-onset depression. The latest research in modern medicine also provides strong evidence for the correlation between hysterectomy and depression^36^. Hyo^37^ extracted data from the Korean Health Insurance from 2002 to 2013, and they found that women who underwent hysterectomy had higher rates of depression than those who did not undergo hysterectomy. We conducted a multimodal observational study using data from the National Health and Nutrition Examination Survey (NHANES) from 2007 to 2020 and found consistent findings across patterns. The risk of depression was significantly increased after hysterectomy compared with those who did not undergo hysterectomy.

Women are twice as likely to be diagnosed with depression as men, because hormone levels are different in women at different times. Current studies have shown that estrogen can play an antidepressant role by regulating neurotransmitters through estrogen receptors, which affect the hypothalamic–pituitary–adrenal axis, and that the ovaries are the organ that secretes estrogen. It follows that the risk of depression should decrease when hysterectomy is performed but ovaries are preserved. However, Wilson^38^ and Laughlin Tomaso^39^ found that women who underwent hysterectomy with preservation of the ovaries were at higher risk for depression than women who underwent both hysterectomy and bilateral oophorectomy. These are two completely opposite conclusions. Based on the above, the current study explored the effect of four different surgical procedures on depression through regression analysis. We found an interesting result that oophorectomy was not associated with positive depression, but hysterectomy was a risk factor for postoperative depression, and the risk of depression was also increased when both the uterus and ovaries were removed.

The uterus is an endocrine organ. In addition to its local endocrine function, it may also regulate the hypothalamic-pituitary-ovarian (HPO) axis which refers to the complete and coordinated neuroendocrine system composing of the hypothalamus, pituitary gland, and ovary. Each of its links has unique neuroendocrine functions, and they regulate and influence each other to maintain a relatively stable dynamic balance^40^. The pituitary secretes follicle stimulating hormone (FSH), prolactin, and luteinizing hormone (LH) under the regulation of Gonadotropin-releasing hormone (GnRH) secreted by the hypothalamus. FSH, prolactin, and LH can act on the ovary and all three participate in the negative feedback regulation of the HPO axis. Studies have shown a tendency to increase FSH levels after Oophorectomy and the opposite for E2 levels^41^. FSH levels are associated with negative emotions such as perimenopausal depression^42^, and women with rapidly rising FSH levels are more likely to experience depressive symptoms^43^, while lower FSH levels are associated with reduced depressive symptoms^44^. Disruption of LH and estrogen regulation after Hysterectomymay be the main mechanism contributing to the increased risk of depression. Similarly, because of estrogen’s role in regulating mood and cognitive function, the sudden drop in estrogen levelsdue to Oophorectomy would presumably increase the incidence of depression. But the study had found that postmenopausal oophorectomy did not affect the incidence of depression. This is consistent with the results of the present study that Oophorectomy-alone was not associated with depression. This may be due to the fact that the age of the sample with Oophorectomy-alone in this study was basically close to menopause. The most significant change around menopause is the decline in ovarian function, which is no longer able to affect hormone levels. In addition, removal of the uterus can cause them to stop believing that they are fully female. This affects their self-confidence and self-worth level^45^, leading to mental health problems. Oophorectomy may exacerbate this psychological burden, which is consistent with the results of this study. Hysterectomy alone is a risk factor for depression, but simultaneous hysterectomy of the ovaries further increases the risk of depression.

The study also found that women after hysterectomy had more depressive symptoms, mainly including little interest, feeling down and trouble concentrating, but not more severe symptoms such as self-denial, slow movement or speech, suicidal tendencies and behaviors. That is, it is associated with depressed mood and somatic symptoms. However, it was not related to slow thinking, decreased volitional activity and cognitive impairment. Pay attention to female mood and somatic symptoms, and positive psychological intervention will improve the rehabilitation effect after hysterectomy^46^.

Therefore, when hysterectomy has become an established fact, but in the case of opportunistic adnexectomy, it is necessary to retain the patient's adnexa as much as possible to reduce the risk of depression. Opportunistic adnexectomy refers to the implementation of oophorectomy and salpingectomy without known indications, such as ovarian lesions, hereditary ovarian cancer syndrome, etc.^47^.

Reviewing our study, there are still some limitations. First, because of its cross-sectional design, it was not possible to determine whether depression was present before the hysterectomy occurred. Second, although this study used a control group matched for demographic factors and several medical histories, even though propensity score methods were used, residual and unmeasured confounding is still possible in this study. The development of depression may be affected by the differences in personality and mentality of each respondent, including preoperative psychosocial status, perioperative pain and postoperative infection.

This study has several strengths. The NHANES data provide us with a unique opportunity to examine the association between hysterectomy and depressive symptoms in this multi-ethnic, representative sample of the population in the United States. Second, to explore the association, we specifically considered the association of hysterectomy with each depressive symptom. Most importantly, we explored the effect of different types of surgical procedures on depression positivity.

## Conclusion

Women who have had a hysterectomy are at higher risk of depression than women who have not had a hysterectomy, and this risk may be exacerbated if the uterus and ovaries are removed. When clinically appropriate, surgeons should try to preserve the patient's ovaries.

## Data Availability

The data that support the findings of this study are available, but restrictions apply to the availability of these data, which were used under license for the current study, and so are not publicly available. Data are however available from the authors via the email address ginayyh@163.com upon reasonable request and with permission of us.
